# Sex Differences in Dysfunctional Movements and Asymmetries in Young Normal Weight, Overweight, and Obese Children

**DOI:** 10.3390/children8030184

**Published:** 2021-03-01

**Authors:** Pat R. Vehrs, Haley Barker, Misea Nomiyama, Zachary Vehrs, Miklόs Tόth, Martina Uvacsek, Ulrike H. Mitchel, Aaron W. Johnson

**Affiliations:** 1 Department of Exercise Sciences, Brigham Young University, Provo, UT 84045, USA; hailey.barker25@gmail.com (H.B.); misae.nomiyama@gmail.com (M.N.); zacharyvehrs@gmail.com (Z.V.); rike@byu.edu (U.H.M.); wayne_johnson@byu.edu (A.W.J.); 2 Department of Health Sciences and Sport Medicine, University of Physical Education, 1123 Budapest, Hungary; tothmik1@hotmail.com (M.T.); uvacsek.martina@tf.hu (M.U.)

**Keywords:** obesity, motor competence, motor performance, functional movement screen

## Abstract

This study evaluated overall performance on the functional movement screen (FMS), prevalence of asymmetries and dysfunctional movements, and the relationship between measures of adiposity and the FMS score. Methods: Ninety-four (53 boys; 41 girls) 10–12-year-old children in Hungary and Germany who were participating in daily physical education performed the FMS. The mean FMS score in girls (14.1) was significantly higher than in boys (12.9). Individual test item scores were similar, except girls scored higher on the straight-leg raise. Most children (55% of boys, 68% of girls) presented with at least one asymmetry and 72% of boys and 76% of girls had at least one dysfunctional score. Measures of adiposity were negatively correlated to performance on all test items. Underweight and normal weight children performed significantly better on the FMS than overweight and obese children. Sex differences and the high prevalence of asymmetries and dysfunctional scores should be interpreted with caution since they may be due to dynamic changes in strength, proprioception, balance, and motor control that occur as part of growth and involvement in activities. Nevertheless, the high prevalence of asymmetries and dysfunctional scores indicate that most children have movement limitations.

## 1. Introduction

Assessments of physical fitness and motor skills can be used to inform and educate participants about physical activity, exercise, and movement competencies. A recent call for research suggested that physical fitness test items need to measure a broad range of fitness as well as dysfunction [1]. Functional movement can be defined as the ability to produce and maintain a balance between mobility and stability along the kinetic chain while performing fundamental movements with accuracy and efficiency [2]. Functional movements are a complex interplay of cognitive, perceptual, proprioceptive, and motor functions that involve muscular strength and endurance, flexibility, coordination, and balance [2]. Movement assessments should consider limb asymmetries, limitations in range of motion, proprioceptive deficits, posture, and pain [3].

One tool that can be used to assess movement patterns in children that also has educational value is the functional movement screen (FMS). Details of the 7 test items (deep squat, hurdle step, in-line lunge, shoulder mobility test, active straight-leg raise, trunk stability push-up, rotary stability test) included in the FMS are well described elsewhere [4,5,6]. Five of the test items (hurdle step, in-line lunge, shoulder mobility test, active straight-leg raise, rotary stability test) are bilateral tests performed on the left and right sides to assess the presence of asymmetries. The test items of the FMS permit the observation of mobility, stability, balance, and functional and dysfunctional movement patterns [5,7].

Although most of the research on the FMS has been done in adults, there is an emerging body of evidence indicating the use of the FMS in children. Previous studies have evaluated the movement competencies of youth in a variety of sports, including secondary school aged athletes [8], soccer [9,10,11,12,13,14,15], Australian football [16], football [15,17], baseball and lacrosse [17], cross country, swimming, tennis, and volleyball [15], and young Olympic athletes [18]. Studies have been conducted in other countries, including Hungary [18], countries of the UK [9,10,13,19,20,21], and Moldova [4]. Differences in the FMS score between pre-pubescent and post-pubescent children have been reported in one study [22]. As some studies included only male participants [9,10,11,12,13,16,17,23,24,25,26], there is a general lack of comparison of performance on the FMS between boys and girls. Five studies have reported no significant differences in the FMS scores between boys and girls [4,18,19,20,22], while two studies have reported that males perform slightly better than their female counterparts [8,27]. The influence of adiposity on FMS test scores is under-reported in the literature. Two studies report a negative association between the FMS score and BMI by age categories and that normal weight children perform significantly better on the FMS than their overweight or obese counterparts [19,20]. Inclusion of other measures of adiposity, such as percent body fat could be informative. Few studies report the prevalence of asymmetries in the bilateral tests and the number of dysfunctional scores (individual test score of 1). For example, Mitchel et al. [4] reported that 64% of boys and 61% of girls had at least one asymmetry. Similar results have been reported in male participants in three other studies [16,17,28]. Although the number of dysfunctional scores varies between individual test items [10,25], up to 85% of children have dysfunctional scores [28].

We undertook this study to investigate asymmetries, dysfunctional scores, sex differences in individual test items and the FMS score, and the relationship between test scores and measures of adiposity in school-aged children who participate in daily physical education. Based on previous literature, we hypothesize that left-to-right asymmetries and dysfunctional scores are common in children, there are no significant sex differences in the FMS score, and increased adiposity adversely affects performance on the FMS.

## 2. Materials and Methods

### 2.1. Participants

Participants included 94 white youth (53 boys; 41 girls), 10–12 years of age enrolled in schools in Germany (Johann-Heinrich-Pestalozzi-Schule; *n* = 19) and Hungary (Semmelweis Egyetem Gyakorlo Altalanos Iskola Gimnazium; *n* = 75). All children were enrolled in daily physical education. Data were collected during the school day at each of the schools. Participating children provided consent to participate in the study and at least one parent provided parental permission for their child to participate in the study. This study was reviewed and approved by the Institutional Review Board at Brigham Young University and appropriate administrations at both schools. 

### 2.2. Physical Measurements

Body mass index (BMI; kg·m-2) and waist-to-hip ratio were calculated from measured values of height (cm), body mass (kg), waist and hip circumferences (cm). Body weight was classified as underweight (<5th percentile), normal weight (5th to <85th percentile), overweight (85th to <95th percentile), or obese (≥95th percentile) based on the BMI-for-age growth charts for children suggested by the Centers for Disease Control (CDC) [29]. Waist and hips circumferences were measured at the narrowest part of the waist or at the level of the naval and the widest circumference of the hip as viewed from the side, respectively. Three measurements were taken at each location and averaged. Body composition was estimated using a handheld bioelectrical impedance device (OMRON HBF306, OMRON Healthcare Inc., Vernon Hill, IL, USA). The OMRON device functions in one of two modes: normal (10–60 years of age) and athlete (active individuals 18–60 years of age). Based on the age criteria of the two modes, the percent body fat (%BF) of the participants in this study was estimated using the normal mode. While standing with their feet slightly apart, the participant held the OMRON grip electrodes with the device in front of their body with their arms fully extended and parallel to the floor. The %BF was recorded from the device’s digital display. 

### 2.3. Functional Movement Screen

The 7 FMS tests were set up in stations. Under the direction and supervision of certified FMS personnel, each child was instructed on how to perform each test by a trained and experienced investigator. As the individual tests were organized in stations with an investigator assigned to each station, children received consistent instruction and cues for performing the tests. Each child performed the 7 FMS tests while being videotaped from the front and side for later review. Details of the 7 items included in the FMS have been described elsewhere [4,5,6]. All items of the FMS (deep squat, hurdle step, in-line lunge, shoulder mobility test, active straight leg raise, trunk stability push-up, rotary stability test) were performed three times. Five of the test items (hurdle step, in-line lunge, shoulder mobility test, active straight leg raise, rotary stability test) were performed on the left and right sides of the body and scored independently. At a later time, two trained and experienced raters reviewed all videos and scored each FMS test for each participant individually. The four-point system was used to score each trial. A score of “3” represents the performance of the correct movement pattern; a score of “2” indicates the involvement of compensatory movements to perform the exercise; and a score of “1” indicates that the movement is incomplete. In trials where participants reported feeling pain, a score of “0” was recorded. In the five bilateral tests, scores for both sides were recorded to note any asymmetries and the lowest score recorded for either the left or right sides was used as the score for the test item. The scores for all 7 tests given by the two raters were compared and in the case of inconsistency; the video was reviewed until the two raters came to a consensus. The sum of the scores for all 7 test items was recorded as the FMS score (maximal score of 21 possible points).

### 2.4. Data Analysis

This study used an observational cross-sectional design. Data were analyzed using SPSS v24 (IBM Corp., Armonk, NY, USA). Sex differences in age, height, body mass, BMI, BMI by age percentile rank, waist and hip circumferences, wait-to-hip ratio, predicted percent body fat, average scores on the bilateral tests, and the FMS score were determined using a one-way analysis of variance. We used Bonferroni’s method for correcting the p value to reduce type I error because of multiple comparisons. The total number of asymmetries, the number of asymmetries for each bilateral test, and the number of dysfunctional scores (score of 1) were compiled. A correlation matrix between individual test items, age and measures of body dimensions was computed. To determine the influence of age, gender, height, body mass, waist-to-hip ratio, BMI, BMI by age percentile rank, and %BF on performance on the FMS, a stepwise forward and backward regression analyses was performed using the FMS score as the dependent variable.

## 3. Results

Descriptive information about the participants in this study are shown in Table 1. There were no significant differences in any of the variables measured between boys and girls. Based on the BMI by age percentiles, 4 participants were classified as underweight, 66 were classified as normal weight, 12 were classified as overweight, and 11 were classified as obese.

The ranges of FMS scores for boys (FMS score 9–18) and girls (FMS score 10–18) were similar. The median score for boys was 13 and the median score for girls was 14. The scores on individual test items and the FMS score are shown in Table 2. Most scores were similar between boys and girls, however girls scored significantly higher on the straight leg raise and the total FMS score. For both boys and girls, the highest average score on an individual FMS test was on the shoulder mobility test and the lowest score was on the trunk stability pushup.

Twenty-four boys (45%) and 13 girls (32%) had no asymmetries, whereas 55% of boys and 68% of girls presented with at least 1 asymmetry (Table 3). Among boys, the greatest number of asymmetries occurred on the shoulder mobility test (32%), followed by the active straight leg raise (30%), rotary stability test (19%), inline lunge (14%), and the hurdle step (5%). For girls, the greatest number of asymmetries occurred in the rotary stability test (30%), followed by the inline lunge (21%), hurdle step (19%), shoulder mobility test (16%), and the active straight leg raise (14%). Both boys (72%) and girls (76%) had the greatest number of dysfunctional scores (score = 1) on the trunk stability pushup test (Table 4).

Generally, the individual FMS test item scores and the total FMS scores were negatively correlated to measures of adiposity in both boys and girls (Table 5). The negative correlations between the total FMS score and BMI (−0.305, −0.423), BMI by age percentile (−0.303, −0.425), and %BF (−0.584, −0.371) in boys and girls, respectively, were significant (Table 5). When participants in the study were categorized according to their BMI by age percentile rank, the data indicate that underweight (14.2 ± 0.9) and normal weight (13.9 ± 2.1) participants performed significantly better on the FMS than overweight (12.1 ± 2.0) or obese participants (11.8 ± 1.4). The FMS score for overweight children was not significantly different from that of obese children. The regression analysis revealed that the %BF was the strongest predictor of the FMS score (R^2^ = 0.179, *p* = 0.0001). Adding sex to the regression increased the R^2^ value to 0.275 (*p* = 0.001) and adding the BMI by age percentile rank increased the R^2^ to = 0.345, *p* = 0.009). Age, height, body mass, BMI, and waist-to-hip ratio were excluded from the regression equation.

## 4. Discussion

This study reports asymmetries, dysfunctional scores, sex differences in FMS scores, and the relationship between test scores and measures of adiposity. The results of this study reveal a sex difference in the FMS score, a concerning prevalence of asymmetries and dysfunctional scores on individual test items and the adverse effects of adiposity on the FMS score.

The overall mean FMS score in this study of 10–12-year-old children was 13.4 ± 2.1 (Table 2) but this varied by sex and BMI category. Our data concur with those of Duncan et al. [20] who report a mean FMS score of 13.2 in 7–10-year-olds that also varied with weight status. Other studies have reported higher mean FMS scores. For example, a group of 8–11-year-old school children in Moldova had a mean FMS score of 14.9 [4], 10–17-year-old school children in India had a mean score of 14.6 [27], and the mean FMS score in a group of seventh- and eighth-grade boys and girls was 15.48 [28]. Overall mean FMS scores (combined boys and girls) reported in the literature should be interpreted with caution as it masks differences in performance on the FMS between sexes, across the age span, and between normal weight, overweight, or obese children.

### 4.1. Sex Differences in the FMS Score

Some studies do not report the sex of the participants [14,21], do not report the FMS scores by sex [18], or do not report statistical significance of sex differences in the FMS score [15,19]. In this study, girls (14.1 ± 1.8) had a significantly higher FMS score than boys (12.9 ± 2.2) (Table 2). Duncan and Stanley [19] reported higher FMS scores in 10- to 11-year-old girls (14.5 ± 2.8) than in boys (13.5 ± 3.4). Although Abraham et al. [27] reported significantly higher FMS scores in 10- to 17-year-old boys (14.93 ± 2.6) than in girls (14.17 ± 2.24), the difference was less than one point. Anderson et al. [8] also reported significantly higher FMS scores in athletic 13- to 18-year-old boys (15.3 ± 2.1) than in girls (13.8 ±1.8). On the contrary, several studies have reported similar FMS scores in athletic boys and girls between 7–18 years of age [4,15,20,22,30]. 

There are obvious differences in participant demographics, specifically age or fitness level between this and previous studies that make it difficult to compare results between studies. For example, in this study and that of Duncan et al. [19], non-athletic participants were 10 to 12 years of age. In the studies that reported similar FMS scores between boys and girls, the participants were either younger [4,20] or older [15,22]. Some studies include athletes in their pool of participants [8,15,22]. Studies that report small or no sex differences in the FMS score suggest that the combined mean FMS score can be used as the normative value for the FMS [27]. On the contrary, data from studies that do report sex differences in the FMS might imply the need to have sex-specific norms. Nevertheless, both extrinsic (age, sex, maturation level, and fitness level) and intrinsic factors (muscle activation, neuromuscular control, and core stability) influence movement quality and therefore the FMS score [8]. Many of these factors are highly dynamic and trainable, suggesting that boys and girls of equal extrinsic and intrinsic development would perform similarly on the FMS. Thus, the sex differences in the FMS in this and other studies [8,19] may reflect differences in physical activity or fitness levels, growth, or maturation. Sex differences in the FMS clearly need further evaluation. 

### 4.2. Sex Differences in Individual FMS Test Items

In this study, boys and girls performed equally as well on all FMS test items, except for the active straight leg raise test on which the girls performed significantly better (Table 2). The active straight leg raise is a complex assessment of dynamic hip mobility, extensibility of the hamstrings, core stability, and motor control, as the participant lifts one leg while keeping the opposing leg in its set position. Previous studies in children have not reported sex differences in the active straight leg raise. The sex differences observed in this study are likely due to factors that contribute to core strength, hip mobility, and motor control, such as the type and amount of physical activity the child normally engages in. In this study, we did not include measures such as the traditional sit-and-reach test, that may have helped explain the sex differences in the active straight leg raise.

Previous studies also report sex differences in the performance of individual FMS test items. In an older group (14–18 years) of secondary school athletes, boys performed better on the trunk stability push-up test and the inline lunge [8]. In a group of 10–17-year-old children, boys performed better on the trunk stability push-up, inline lunge, and rotary stability tests [27]. Since various factors may contribute to sex differences in individual FMS test items, sex differences are likely to become less apparent with improved muscle strength and activation, neuromuscular control, core stability, flexibility and joint mobility. This is evidenced in a group of elite junior athletes 16–20 years of age, in which there were no sex differences in any of the individual FMS test items [18]. As noted above, because intrinsic and extrinsic factors that affect performance on the FMS are highly dynamic, sex differences in individual test items should be interpreted carefully.

### 4.3. Measures of Adiposity and the FMS Score

In this study, almost all individual FMS test item scores and the total FMS score were negatively correlated to indicators of obesity (i.e., BMI, BMI percentile rank, and percent body fat). Children who were either underweight (14.2 ± 0.9) or normal weight (13.9 ± 2.1) had significantly higher FMS scores than children who were overweight (12.1 ± 2.0) or obese (11.8 ± 1.4). Our data concur with those of two previous studies in which the FMS score in 10 to 11 year old [19] and 7–10 year old [20] children was negatively correlated to BMI and underweight and normal weight children had a significantly higher total FMS score (15.5 ± 2.2; 14.7 ± 2.4) than overweight or obese children (10.6 ± 2.1; 9.0 ± 1.7), respectively. To the best of our knowledge, this is the first study in children to assess body composition (i.e., percent body fat; %BF) as a measure of adiposity. In this study, %BF had the highest correlation between any of the measures of adiposity and the total FMS score (Table 5). The correlation between %BF and individual test items varied between the test items and sex, suggesting that body composition affects individual test items differently in boys and girls. One possible explanation is that total body fat or distribution of body fat may have a greater (or lesser) effect on tests depending on the strength, balance, or motor control requirements of the test or if the movement involves primarily the upper body or lower body.

In this study, 26% of the participants were overweight or obese. The regression analysis of the data in this study indicated that measures of adiposity (%BF and BMI by age percentile) were the strongest predictors of performance on the FMS. A previous study also reported that BMI by age percentile was the strongest predictor of the FMS score [19]. Previous reports indicate that functional limitations are a consequence of excess body weight and that overweight and obesity alters functional movements in children [31,32]. The impact of excess body weight on functional movements could be related, in part, to foot structure and function, inadequate muscular strength and power, and walking gait [31,32]. 

Because functional limitations may have existed prior to becoming overweight or obese, the correlation between BMI and the total FMS at a single point in time does not imply cause and effect. Nevertheless, overweight and obese children present with functional limitations as measured by the FMS. These consequences can lead to maladapted movement patterns, musculoskeletal discomfort, pain, fatigue, disturbance in proprioception, and poor balance [31,32]. Children with functional limitations are less likely to be physically active and will not develop fundamental movement patterns as well as children who do not have functional limitations [19,31]. The effects of overweight or obesity on joint loading coupled with limitations in functional movement patterns may lead to orthopedic abnormalities later in life and prevent the attainment of the health benefits of regular participation in physical activity [32]. On the contrary, children who do not have functional limitations are more likely to enjoy physical activity and therefore develop proficiency in fundamental movement patterns and greater physical self-efficacy [19]. Physical education programs in public and private schools are a reasonable venue to help curb the obesity epidemic, increase physical activity among children, and improve quality of movement. Physical education programs may have the greatest potential impact since they could be inclusive of all children. Assessments in physical education programs are common. The FMS could be used as an educational tool to assess and inform children of aspects of well-being that are not evaluated in traditional physical fitness tests. As with other assessments of physical fitness, the FMS can be used as a teaching tool to encourage changes in behavior but should never be used to shame or guilt children about poor performance or body size. 

### 4.4. Effects of Age on the FMS Score

In this study of 10–12-year-old children, there was a weak positive correlation between age and the FMS score in girls (0.242) and boys (0.053). The weak correlation may simply be due to the narrow age range of the participants. Nevertheless, a previous study involving children 10–17 years of age reported a weak negative correlation (R = -0.038) between age and the FMS score [27]. To the contrary, results from previous studies indicate that older children generally perform better on the FMS than younger children [10,22]. Higher FMS scores in older children suggest that the competency of fundamental movement patterns is highly dynamic and is influenced by changes in muscle strength, muscle activation, proprioception, neuromuscular control, balance, coordination, core strength, mobility and flexibility during the normal growth and maturation periods of childhood and adolescence [8,22]. This may also be related to increased participation in various recreational activities and sports that develop these characteristics. The generalizability of a single normative FMS score across the age span of childhood and adulthood is not appropriate. 

### 4.5. Asymmetries and Dysfunctional Scores

Assessing asymmetries in the FMS is important because having asymmetrical movement patterns in two or more individual tests items increases the risk of injury [23]. In this study, 60% of participants (55% of boys and 68% of girls) presented at least 1 asymmetry (Table 3). Our data are similar to those of a previous study reporting a 64% and 61%prevalence of at least one asymmetry in 8–11-year-old boys and girls, respectively [4]. Three studies that included 13–18-year-old male participants [16,17,33] reported a 63% to 68% prevalence of at least one asymmetry. The greatest number of asymmetries in this study occurred on the shoulder mobility test for boys and the rotary stability test for girls. Mitchell et al. reported that both boys and girls had the greatest number of asymmetries when performing the hurdle step [4]. Two studies in boys reported the greatest number of asymmetries in the shoulder mobility test [16,33]. 

A dysfunctional score (score = 1) on the FMS indicates that the participant was unable to complete the movement as instructed. In this study, 86 of the 94 children (92%) presented a dysfunctional score on at least one FMS test item, which is in agreement with the 93% of male cricket players previously reported [25]. These data are higher than the 68% of high school athletes [17] and 85% of 7th and 8th grade physical education students [28] with dysfunctional scores on at least one test item. In this study (Table 4), and in a study of high school swimmers [30], the trunk stability pushup test had the greatest number of dysfunctional scores. Other studies reported the greatest number of dysfunctional scores on the deep squat [16,26,30], rotary stability [25], shoulder mobility [26], and trunk stability pushup [30]. Some studies report that dysfunctional scores were recorded in every individual test item [16,25,30].

Asymmetries and dysfunctional scores on the FMS in children should be interpreted with caution since some asymmetries may be expected when proprioception, balance, strength, and motor control are still developing. In addition, asymmetries may be related to participation in activities that have asymmetrical movement patterns, such as sports that involve kicking or throwing [33,34]. Differences in the individual FMS test items with the highest number of dysfunctional scores may reflect differences in physical development and sports participation. The FMS could be a useful tool to assess the presence of asymmetries, determine underlying causes, and track changes over time.

### 4.6. Limitations of This Study and Future Directions

Although we believe that the participants of this study represent the larger population of boys and girls of this age in Hungary and Germany, future studies could involve a cohort of children across a wider age range and number of schools. In this study we did not collect data from other assessments of physical fitness or motor skills. Although growth and development affect motor competencies, this study did not measure or estimate maturation.

Future studies can be more transparent in the reporting of data, including information about the sex and age of the participants, measures of body size or body composition, prevalence of asymmetries and dysfunctional scores, and sex differences in the FMS score as well as individual test items. To further evaluate the relationship between adiposity and performance on the FMS, future studies would need to include adequate representations of each of the BMI by age weight categories for both boys and girls. Comparing the FMS score, asymmetries, and dysfunctional scores in inactive, physically active, and athletic boys and girls in different age groups would help clarify the influence of various factors on performance on the FMS. Given the high prevalence of asymmetries and dysfunctional scores, implementation and evaluation of interventions designed to improve movement competency in children are warranted.

## 5. Conclusions

This study investigated the performance of young boys and girls on the FMS. The four primary findings were: (a) the girls in this study had higher overall FMS scores than boys, (b) the majority of children demonstrated at least one asymmetry and at least one dysfunctional score, (c) performance on individual test items was negatively correlated to indicators of obesity, and (d) children who were classified as underweight or normal weight performed significantly better on the FMS than overweight and obese children. The sex differences in the FMS score and presence of asymmetries and dysfunctional scores in young children should be interpreted in the context of the dynamic changes in strength, proprioception, balance, and motor control that occur as part of normal growth, development and involvement in physical activities. Movement competencies are more likely to develop with appropriate instruction, practice, and feedback [35,36,37]. Fitness professionals and physical educators can use the FMS to identify movement dysfunctions and implement age and developmentally appropriate activities to improve motor competency as part of a physical fitness program. An appropriate use of the FMS would be to monitor changes in individual scores, asymmetries, and dysfunctional scores that occur over time as a result of participation in a well-structured program. 

## Figures and Tables

**Table 1:** Participant Characteristics.

	Boys (*n* = 53)	Girls (*n* = 41)	Combined (*n* = 94)
Age (yrs)	10.4 ± 1.36	10.4 ± 1.1	10.4 ± 1.25
Height (cm)	146.9 ± 10.9	147.9 ± 9.5	147.4 ± 10.3
Body Mass (kg)	40.9 ± 9.5	41.3 ± 10.5	41.0 ± 9.9
Waist Circumference (cm)	66.5 ± 7.4	65.6 ± 9.8	66.1 ± 8.5
Hip Circumference (cm)	78.2 ± 7.9	79.4 ± 9.8	78.7 ± 8.8
WHR	0.85 ± 0.05	0.82 ± 0.05	0.84 ± 0.05
BMI (kg/m^2^)	18.8 ± 3.1	18.6 ± 3.4	18.7 ± 3.2
BMI (age percentile)	61.1 ± 30.3	55.1 ± 29.2	58.5 ± 29.8
Body Fat (%)	25.1 ± 8.3	25.9 ± 5.5	25.5 ± 7.2

WHR = waist-to-hip ratio. BMI = body mass index. No statistical significance between boys and girls in any of the variables.

**Table 2:** Scoring of individual test items of the functional movement screen (FMS).

	Boys (*n* = 53)	Girls (*n* = 41)	Combined (*n* = 94)
Deep Squat	1.6 ± 0.6	1.8 ± 0.7	1.7 ± 0.6
Hurdle Step	2.0 ± 0.2	2.2 ± 0.4	2.1 ± 0.3
Right	2.0 ± 0.2	2.2 ± 0.4	2.1 ± 0.3
Left	2.0 ± 0.2	2.2 ± 0.4	2.1 ± 0.3
Inline Lunge	2.1 ± 0.4	2.1 ± 0.4	2.1 ± 0.4
Right	2.1 ± 0.4	2.2 ± 0.5	2.1 ± 0.4
Left	2.1 ± 0.5	2.3 ± 0.4	2.2 ± 0.5
Shoulder Mobility	2.4 ± 0.8	2.7 ± 0.4	2.6 ± 0.7
Right	2.6 ± 0.7	2.8 ± 0.4	2.7 ± 0.6
Left	2.5 ± 0.8	2.8 ± 0.4	2.6 ± 0.7
Active Straight Leg Raise	1.7 ± 0.7	2.3 ± 0.7 *	2.0 ± 0.8
Right	1.8 ± 0.7	2.4 ± 0.6 *	2.1 ± 0.7
Left	1.8 ± 0.7	2.3 ± 0.7 *	2.0 ± 0.8
Trunk Stability Pushup	1.4 ± 0.6	1.3 ± 0.6	1.3 ± 0.6
Rotary Stability	1.6 ± 0.5	1.7 ± 0.5	1.6 ± 0.5
Right	1.7 ± 0.5	1.9 ± 0.6	1.8 ± 0.5
Left	1.6 ± 0.5	1.8 ± 0.6	1.7 ± 0.5
Total FMS Score	12.9 ± 2.2	14.1 ± 1.8 *	13.4 ± 2.1

* = significant difference between boys and girls.

**Table 3:** Prevalence of asymmetries in boys and girls.

	Boys (*n* = 53)	Girls (*n* = 41)	Combined (*n* = 94)
No Asymmetries	24	13	37
One Asymmetry	22	16	38
Two Asymmetries	6	9	15
Three Asymmetries	1	3	4

**Table 4:** Distribution of dysfunctional scores for each test.

	Boys (*n* = 53)	Girls (*n* = 41)	Combined (*n* = 94)
Deep Squat	22	13	35
Hurdle Step	0	0	0
Inline Lunge	3	1	4
Shoulder Mobility	10	0	10
Active Straight Leg Raise	23	6	29
Rotary Stability	21	13	34
Trunk Stability Pushup	38	31	69
Total	117	64	181

A dysfunctional score is a score of one (1) on a test item.

**Table 5:** Correlation matrix between anthropometric measures and FMS scores.

	Age	kg	cm	BMI	BMI%	WHR	%BF
Squat							
Boys	0.203	−0.189	−0.081	−0.206	−0.176	−0.085	−0.404 *
Girls	0.037	−0.332 *	−0.133	−0.377 *	−0.277	−0.362 *	−0.262
Hurdle Step							
Boys	0.308	0.276	0.399 *	0.009	−0.014	−0.194	−0.205
Girls	−0.232	−0.330 *	0.090	−0.385 *	−0.433 *	0.134	−0.181
Inline Lunge							
Boys	0.028	−0.075	0.031	−0.125	−0.153	−0.013	−0.266
Girls	0.411 *	0.057	0.199	−0.061	−0.087	−0.058	−0.243
Shoulder Mobility							
Boys	0.033	−0.100	0.082	−0.213	−0.184	−0.006	−0.255
Girls	−0.009	−0.223	−0.012	−0.316 *	−0.285	−0.011	−0.082
Active Straight Leg Raise							
Boys	0.078	−0.302 *	−0.100	−0.333 *	−0.361 *	0.115	−0.296
Girls	−0.059	−0.188	−0.066	−0.327 *	−0.322 *	0.083	−0.198
Rotary Stability							
Boys	0.323	0.088	0.193	−0.068	−0.100	−0.153	−0.454 *
Girls	0.131	0.007	0.133	−0.105	−0.182	−0.135	−0.047
Trunk Stability Pushup							
Boys	0.152	−0.048	0.016	−0.088	−0.065	−0.125	−0.365 *
Girls	0.011	−0.018	0.001	−0.014	−0.003	0.040	−0.296
Total FMS Score							
Boys	0.242	−0.170	0.064	−0.305 *	−0.303 *	−0.083	−0.584 *
Girls	0.053	−0.274	0.044	−0.423 *	−0.425 *	−0.098	−0.371 *

kg = body mass (kg), cm = height (cm), WHR = waist-to-hip ratio, %BF = percent body fat. * = significant correlation (*p* < 0.05).

## Data Availability

The data presented in this study are available upon request from the corresponding author. The data are not publicly available.
